# Methodology for using a Bayesian nonparametric model to uncover universal patterns in color naming

**DOI:** 10.1016/j.mex.2021.101572

**Published:** 2021-11-02

**Authors:** Kirbi Joe, Maryam Gooyabadi

**Affiliations:** Institute for Mathematical Behavioral Sciences, University of California, Irvine, USA

**Keywords:** Machine Learning, Data Analysis, Mixture Model, Applied Mathematics, World Color Survey, Color Naming, Universality

## Abstract

Language is an integral part of society which enables communication among its members. To shed light on how words gain their meaning and how their meaning evolves over time, color naming is often used as a case study. The color domain can be defined by a physical space, making it a useful concept for studying denotation of meaning. Though humans can distinguish millions of colors, language provides us with a small, manageable set of terms for categorizing the space. Partitions of the color space vary across different language groups and evolve over time (e.g. new color terms may enter a language). Investigating universal patterns in color naming provides insight into the mechanisms that give rise to the observed data. Recently, computational techniques have been utilized to study this phenomenon. Here, we develop a methodology for transforming a color naming data set—namely, the World Color Survey—which is based on constraints imposed by the stimulus space. This transformed data is used to initialize a nonparametric Bayesian machine learning model in order to implement a culture and theory-independent study of universal color naming patterns across different language groups. All of the methods described are executed by our Python software package called *ColorBBDP*.

• Data from the World Color Survey is transformed from its original format into binary features vectors which can be given as input to the Beta-Bernoulli Dirichlet Process Mixture Model.

• This paper provides a specific application of Variational Inference on the Beta-Bernoulli Dirichlet Process Mixture Model towards a color naming data set.

• New mathematical measures for performing post-cluster analyses are also detailed in this paper.

## Graphical abstract

**Figure:**
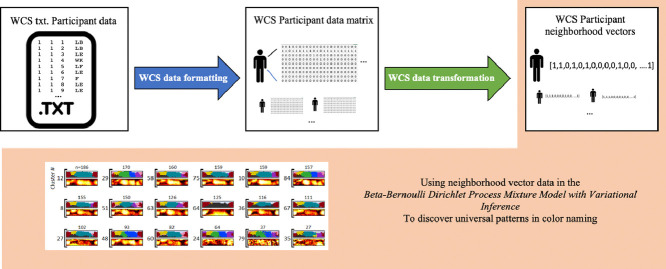

Specification table

**Table:** 

Subject Area	Computer Science
More specific subject area	Bayesian nonparametric models
Method name	*ColorBBDP* (Python Package for implementing the Beta-Bernoulli Dirichlet Process Mixture Model on Color Naming Data)
Name and reference of original method	Beta-Bernoulli Dirichlet Process Mixture Model with Variational Inference Hughes, M. C., & Sudderth, E. (2013). Memoized online variational inference for Dirichlet process mixture models. In Advances in Neural Information Processing Systems (pp. 1133-1141). Ni, M., Sudderth, E. B., & Hughes, M. Variational Inference for Beta-Bernoulli Dirichlet Process Mixture Models.
Resource availability	https://github.com/kirbijoe/colorBBDP

## Introduction

The study of language and its evolution has been a topic long studied in academia. Within this field of study, color has been a particularly useful case study for investigating important features of language because of its property as a physical, quantifiable entity. Since color exists in a tangible space, the color categories of a language can be clearly denoted. That is, the meaning of color terms can be clearly defined and, thus, compared cross-culturally. Studying the commonalities of these color categories across different linguistic groups can identify universal patterns and help uncover the mechanisms which cause languages to develop and evolve over time.

**Fig. 1:**
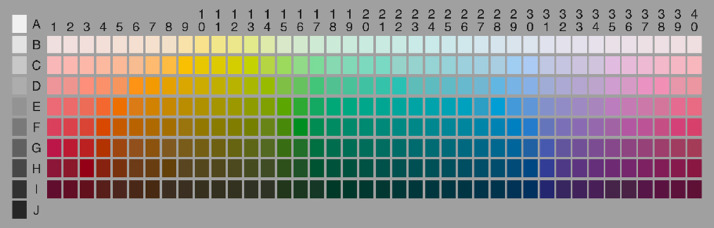
The set of 330 Munsell color chips used in the World Color Survey.

In 1969, Brent Berlin and Paul Kay published their book *Basic Color Terms: Their Universality and Evolution*, which sparked a renewed interest in the study of color naming in academia and gave way to decades of research on this topic. As an extension to their existing work and in an effort to provide empirical evidence of their hypotheses, Berlin and Kay conducted the World Color Survey (WCS) [9]. The data from the WCS was collected from 110 unwritten, monolingual, pre-industrial, tribal languages, with an average of 24 participants per language (∼2,640 participants in total). Participants completed two tasks: the *naming task* and the *mapping task*. In the naming task, participants assigned names to 330 Munsell color chips (see Fig. 1), which were presented one at a time, in a fixed random order. In the *mapping task*, participants were given a color term from their language and were asked to pick a color chip (or set of color chips) from the stimulus set which best exemplified that term. This set of color chips were referred to as *focal colors*. This data set has been widely used to study the properties and evolution of color naming systems.

At the time of their inception, the discussion and analysis of color naming was predominantly based in linguistics and anthropology. In recent years, though, there has been an increase in the application of quantitative methods and approaches to the WCS data, including machine learning technique. The application of machine learning methods to the WCS has been conducted primarily by Brown and Lindsey. They performed the k-means clustering algorithm on both the individual terms used [10] as well as individual participant systems [11]. The clustering of the color terms revealed a constrained set of color categories—easily equated with the English color categories and some composites—with which almost all languages could partition the color space. The use of k-means to cluster the set of participant categorization systems revealed universally occurring *motifs*. These motifs were taken as representations of universal patterns found in the data. Brown and Lindsey concluded that (i) motifs were widespread and present in many unrelated languages, pointing to their universality, and (ii) that there was a surprising level of diversity of motifs within languages. Both of these studies revealed universal properties of the WCS data set, some of which were consistent with the claims of Berlin and Kay [3]. These studies showed the variety of ways machine learning methods could be applied to this data set and their ability to draw conclusions about universality (and even the possible evolutionary trajectory) of color naming systems.

We follow in the same vein as Lindsey and Brown [11] in that we hope to discover possible universal structures through a clustering of participant data. However, our approach extends the previous literature by endeavoring to perform a clustering of the data without any assumptions about the models’ level of complexity. This can be achieved through the use of nonparametric variational Bayesian inference methods to approximate the parameters of a generative, unsupervised infinite mixture model.

## Data set used

This study uses *naming task* data gathered from WCS participants [5]. The data is publicly available at no cost via the survey website (http://www1.icsi.berkeley.edu/wcs/data.html). The *naming task* data can be found in the term.txt file on the project website. The data text sheet consists of a long list of the names participants from all languages assigned to each color chip where each line contains the language number (1 to 110), participant number, chip number (1 to 330), and abbreviation of term used. Supplementary text files contain information on each language, participant details, as well as data from the naming task. Of the 110 languages surveyed, four languages^1^ are omitted from our data set due to data collection and transcription issues. Only the 320 chromatic chips (column B1–I40 in Fig. 1) are included in the data due to their disjointed nature from the 10 achromatic chips. Therefore, all future references “the data” will be to the collection of naming task data for 106 languages (2,552 individuals) on the 320 chromatic chips in the color grid. For our purposes, the WCS data is formatted into a naming matrix for each participant. Each matrix has 320 columns representing the set of color chips (see Fig. 1) and n rows based on number of terms used by the participant. A cell (i,j) given a values of 1 if the participant used name i to name chip j and 0 otherwise. Each column will have exactly one 1 (i.e. a color chip can only have one term).

## Method details

Variational Inference for the Beta-Bernoulli Dirichlet Process Mixture Model (BBDP) [7,13] is employed to uncover universal patterns in the WCS [8]. It combines a Beta-Bernoulli observation model with the Dirichlet Process mixture model. Together, it allows us to cluster binary features vectors of participants without assuming the number of clusters. Model selection is performed by using variational inference methods [4] to estimate the lower bound of the marginal likelihood (i.e. the evidence of the lower bound or ELBO) of the observed data. Doing so first requires transforming the WCS in such a way that is compatible with the model and allows for cross-group comparisons. Our *ColorBBDP* software package transforms the data based on the physical constraints of the stimulus space so that the BBDP can compare participants from across language groups.

### Beta-Bernoulli Dirichlet process mixture model with variational inference

The mathematical formalization of this model, as described below, is also included in the Appendix of Joe and Gooyabadi's paper which reported experimental results from an implementation of this methodology [8].

*Beta-Bernoulli Mixture Model:* Suppose we have data set $X\;=\;\{X_{1},\;...,\;X_{N}\}$, where each observation $X_{i}$ is a binary vector with D dimensions representing D attributes of an observation. An entry $x_{id}=1$ if $X_{i}$ has the attribute *d* and $x_{id}=0$ otherwise. If we let θ be the mean of the Bernoulli distribution, then the Bernoulli likelihood can be written generally as:

$$P\left(x|\theta \right)\;=\theta^{x}{\left(1-\theta \right)}^{1-x}$$

Using this form, the probability density for each observation *X_i_* can then be computed by:

$$P\left(X_{i}|\theta \right)\;=\prod_{d=1}^{D}\theta_{d}^{x_{\mathrm{id}}}{\left(1-\theta_{d}\right)}^{1-x_{\mathrm{id}}}$$

where θ is a D-dimensional vector with entries $\theta_{d}$ for d∈{1,...,D} represent the probability that an observation has the attribute d.

The conjugate prior to the Bernoulli distribution is the Beta distribution with parameters $\beta_{1}$ and$\;\beta_{2}$. Therefore, the prior, P(θ) can be given by the following function:

$$P\left(\theta \right)=\frac{1}{B\left(\beta_{1},\beta_{2}\right)}\theta^{\beta_{1}-1}{\left(1-\theta \right)}^{\beta_{2}-1}$$

where the Beta function $B(\beta_{1},\;\beta_{2})$ serves a normalization constant and *β_1_, β_2_* are shape parameters that determined based on prior beliefs or existing knowledge. We only search the portion of the parameter space where $\beta_{1},\;\beta_{2}\in \left(0,\;1\right)$ because the shape of the beta distribution is biased towards the bounds of its domain, 0 and 1, when $\beta_{1},\;\beta_{2}$ < 1. This behavior is useful when drawing priors for a Bernoulli mixture model.

A Beta-Bernoulli mixture model can be defined by a mixture of K Beta-Bernoulli distributions. In order to identify which of the K distributions each data point $X_{i}$ was drawn from, we introduce a latent variable $Z=\{Z_{1},\;...,\;Z_{N}\}$. For each Zi∈Z, $Z_{i}\;$is a K-dimensional vector which has exactly one entry equal to 1, corresponding to the cluster assignment of $X_{i}$. Each of the Kdistributions in the mixture model has a corresponding weight, represented by $\pi =\{\pi_{1},\;...,\;\pi_{K}\}$, such that $\sum_{k=1}^{K}\pi_{k}=1$. Therefore, the distribution of the latent variable Z conditioned upon its weights π is:

$$P\left(Z|\pi \right)=\prod_{i=1}^{N}\prod_{k=1}^{K}\pi_{k}^{z_{\mathrm{ik}}}$$

and the Bernoulli likelihood can then be formalized as:

$$P\left(X|Z,\theta \right)=\prod_{i=1}^{N}\prod_{k=1}^{K}P{\left(X_{i}\theta_{k}\right)}^{z_{\mathrm{ik}}}$$

**Fig. 2:**
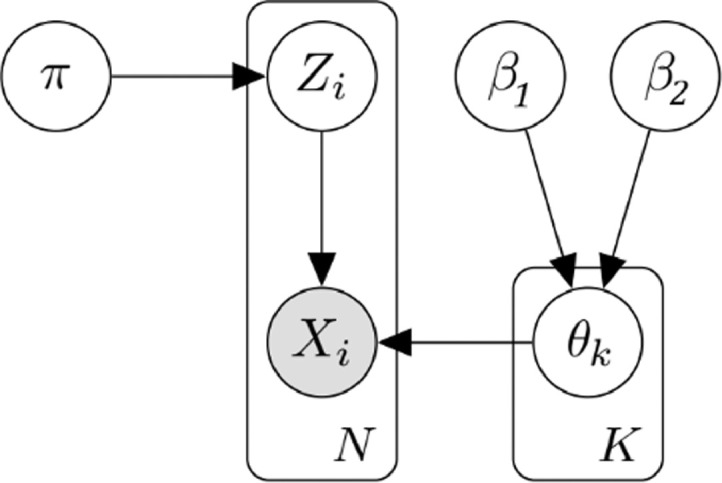
A graphical model of the Beta-Bernoulli mixture model.

Fig. 2 depicts a graphical model representation of the Beta-Bernoulli mixture model.

*Dirichlet Process Mixture Model:* The Dirichlet Process (DP) is a nonparametric prior for infinite, discrete distributions. Therefore, the DP mixture model is able to cluster exchangeable data points without determining the number of clusters *a priori* by assuming an infinite number of latent clusters. For this reason, DP mixture models are synonymously known as infinite mixture models. These processes are commonly used in Bayesian nonparametric methods because they allow the number of clusters to grow as more data points are introduced to the model.

A DP can be thought of as a distribution over distributions. Suppose *G* is a Dirichlet process, then $G∼DP(\alpha ,\;G_{0})$ where $\alpha \in R^{+}$ is called the dispersion parameter and $G_{0}$ is the base probability distribution. Draws from the process G are taken according to the following algorithm:

1. Assume there are $X_{1},\;...,\;X_{N}$ observations and k unique values for the variable K (which represent k clusters present at the time).
2. For observation $X_{i}$, with probability $\frac{\alpha }{N-1+\alpha }$, a new draw is taken from $G_{0}$ (i.e. $X_{i}$ is assigned to a new cluster).
3. With probability $\frac{n_{k}}{N-1+\alpha }$, where $n_{k}$ is the number of observations currently in cluster k, $X_{i}$ joins cluster k.
4. Each observation is iteratively assigned to a cluster until all N observations have been grouped. Cluster assignments are stored in the latent variable Z where $Z_{i}\in \;Z$ is a K-dimensional vector with the k-th element being equal to 1 (corresponding to datum $X_{i}$ being assigned to the cluster k) and all other elements equal to 0.

**Fig. 3:**
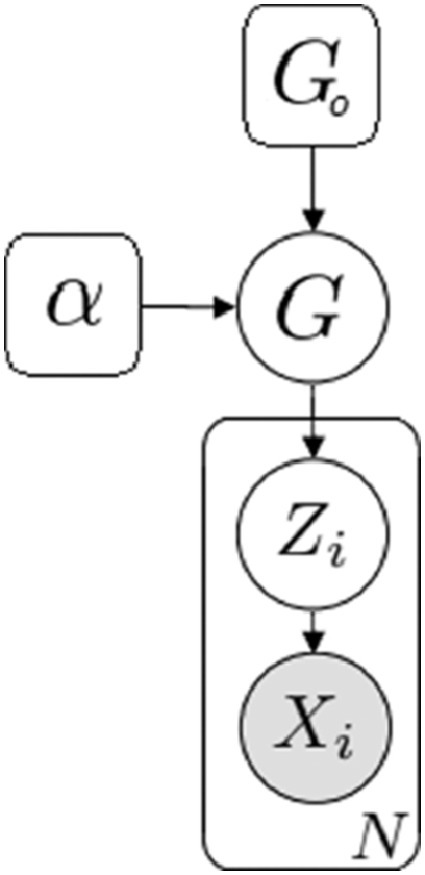
A graphical model of the Dirichlet Process mixture model.

The end result of this process is then a distribution over the partitions of the data X, which serves as a prior over the class assignment vector Z. Some common analogies used to describe the DP are the Chinese Restaurant Process, the Stick-Breaking Construction, and a modified version of Polya's Urn Scheme. A graphical model for the DP mixture model is presented in Fig. 3.

*Variational Inference:* Due to the complexity of the statistical models in Bayesian nonparametric methods, many of the resulting integrals become intractable and thus require other techniques to approximate the parameters of the model. One such family of techniques is called *variational Bayesian inference*. Variational inference can be used as a way to (i) estimate the model's posterior distribution or (ii) to compute an evidence of the lower bound (ELBO), which is then used for model selection. The intuition behind (ii) is that the higher the computed marginal likelihood of a model is, the higher the probability that the data was generated by that model. Therefore, the model with the highest ELBO is selected as the most appropriate model, given the data. In this paper, we use variational inference for the purpose of computing the lower bound of the marginal likelihood.

Given a set of unobserved variables Z and a data set X, the posterior distribution can be approximated by the variational distribution Q: P(Z|X)≈Q(Z). The aim of variational inference is to minimize the distance between the true posterior P(Z|X) and the approximated distribution Q(Z) and thus seeks to find the Q which minimizes this distance. The distance between distributions P and Q is most often formalized using Kullback-Leibler Divergence (KL-divergence), defined as

$$KL\left(Q∥P\right)=\sum_{Z}Q\left(Z\right)\log\frac{Q\left(Z\right)}{P(Z|X)}$$

This function can be rewritten and rearranged to yield

$$\log P\left(X\right)=KL\left(Q∥P\right)-E_{Z}\left[\log Q\left(Z\right)-\log P\left(Z,X\right)\right]=KL\left(Q∥P\right)+L\left(Q\right)$$

The term L(Q) is called the Evidence Lower Bound (ELBO). Maximizing L(Q) will reveal the Q which minimizes the KL-divergence since logP(X)is fixed with respect to Q.

### Transforming world color survey participant data

**Fig. 4:**
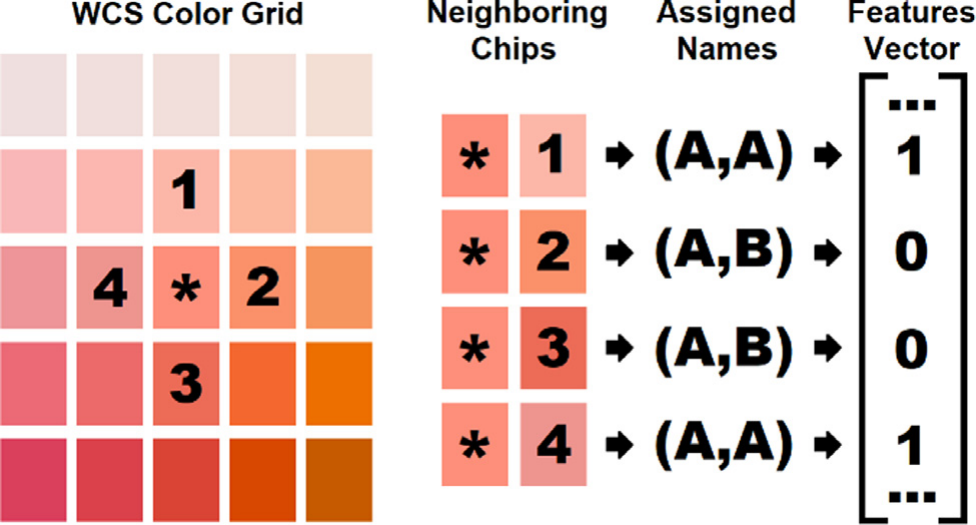
An example of the data transformation process from WCS naming task data to a binary features vector (original figure published in [8]). This transformation is achieved by considering the given names of neighboring color chips. Performing this transformation generates a set of binary features vectors which are comparable with each other, regardless of the language spoken by the participants.

The *ColorBBDP* dissociates the participants from the specific color terms they used by transforming the naming matrix of each WCS participant into an n-dimensional binary features vector. The binary vector is constructed by comparing the names given to neighboring color chips by a single WCS participant. Color chips in the chromatic component of the WCS color grid (chips B1–I40 in Fig. 1) are chosen one at a time to be the *reference chip*, and the name for the *reference chip* is compared to the name of one of its 4 vertically and horizontally adjacent neighbors (or 3 in the case of chips located on rows B and I^2^). Each element of the binary vector represents one of these pairs. The value of an index in the vector is set equal to 1 if the two color chips being compared have the same name and 0 if they have different names (see Fig. 4). This comparison was performed for all possible pairs of neighboring color chips.

Transforming the *naming task* data in this way results in a set of 2,552 data points each with 2,320 binary attributes [8]. In order to prevent the model from needing to parse through excessive amounts of data and since this pairwise judgment is reflexive, redundant pairs are omitted from the features vector; that is, (chip i, chip j) is included in the vector, but not (j,i).

*ColorBBDP* is able to maintain the structure of the original naming schema while abstracting away from their linguistic origin, by drawing on properties of the physical color space. Approaching the problem in this way (i.e. abstracting away from the specific color terms used) allows for a cross-group study of universal patterns where the BBDP clusters participants based on adjacent neighbor-like judgments.

### Model implementation

The model described in this paper is a special case of an infinite mixture model called the Beta-Bernoulli Dirichlet Process Mixture Model, using variational inference for model selection [7,13]. The main advantage of using this model is the ability to perform a clustering without needing to define the resulting number of clusters *a priori*. Infinite mixture models are able to achieve this by assuming an infinite number of latent clusters and then letting the resulting clusters grow as more data is introduced to the model.

There is a Python package called BayesPy [12] that can construct the required models and can perform inference over these models. BayesPy is a tool that implements variational Bayesian inference on conjugate exponential family models. Since our data is binary in nature, we define a Bernoulli likelihood function. The conjugate prior to the Bernoulli distribution is the Beta distribution, resulting in a Beta-Bernoulli observation model. BayesPy approximates infinite dimensional distributions, such as the Dirichlet process, by setting the maximum number of clusters K to a value much higher than the number of expected clusters. We initialize K=100 clusters and consistently find the number of resulting clusters $K^{*}<100$, indicating that the results are driven by the data and not the upper bound [8]. These parameters can be altered in the *ColorBBDP* program.

### Selecting hyperparameters

Though one of the main benefits of implementing a model using Bayesian nonparametric methods is the ability of the model to freely determine parameters' values throughout the training process, these models still contain variables which need to be exogenously determined. These variables are called *hyperparameters*. Several methods are commonly used in order to search the parameter space for set of hyperparameters which will yield the most “optimal” result, such as grid search, random search, Bayesian optimization, and evolutionary optimization [1,2]. We chose to use random search to estimate values for our model's hyperparameters.

**Fig. 5:**
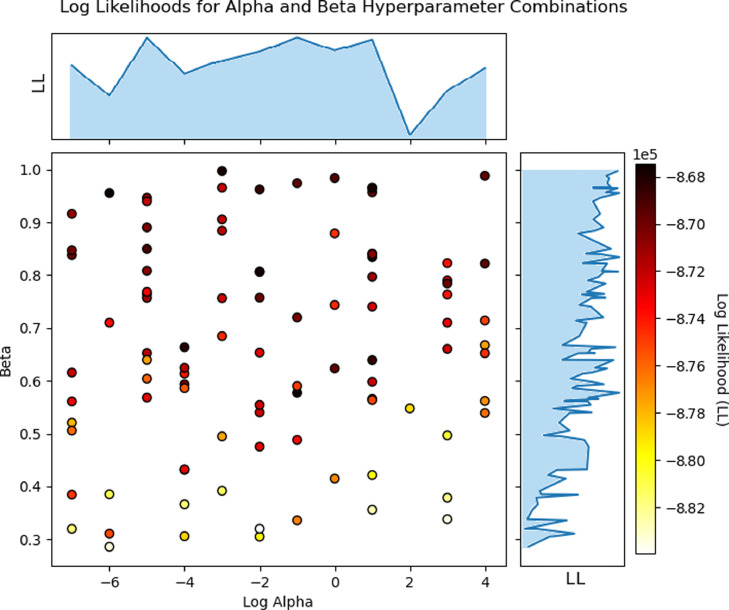
Scatter plot representing 100 random initializations of the BBDP. The x-axis represents the *α* parameter (Dirichlet process concentration parameter) in log units. The y-axis represents the *β* hyperparameter ($\beta_{1},\;\beta_{2}$ of the beta distribution). The darker the color of the data point, the higher the ELBO of the algorithm run using those hyperparameters.

A naive search method is employed instead of one which actively searches for an optimum (e.g. Bayesian or evolutionary optimization) because the more simplistic approach was found to be sufficient for the purposes of this study. Hence, random search is used to search the parameter space for an estimate of the optimum. The optimal parameters are the determined by finding the combination which yields the highest ELBO. Several sets of 100 random initializations were run at a time in an effort to determine general regions of optimality. This revealed a broad pattern. Higher values of β generated higher ELBOs whereas α did not appear to have much of an influence on the value of the ELBO (see Fig. 5). This finding is consistent with the fact that the model is more sensitive to the beta distribution hyperparameters than the Dirichlet process concentration parameter [13]. Therefore, based on the pattern obtained from running multiple set of initializations and precedence from previous literature, the hyperparameters selected for the model were α=1000 and $\beta =\beta_{1}$, $\beta_{2}=0.9$ [8].

### ColorBBDP software features

The *ColorBBDP* software package contains novel methodologies to perform comparisons between individuals and groups as well as graphing functions for an easy visual representation. Further explanation and formalization of the following features are detailed in [8].

*Centroids*: The centroid function provides a singular representation of a group. Centroids are constructed by taking the modal term used by the group's participants for each color chip. By constructing the centroid in this manner, it becomes the representation which minimizes distance from every other member of the group.

*Boundary Heatmaps*: Boundary heatmaps also provide singular representations of a group, but provide a more informative figure by revealing the underlying strength of the category partitions. Strength is defined as the level of agreement among the group over the partition depicted by the centroid. By considering both the group centroids and boundary heatmaps, similar modal maps are able to be distinguished through varying regions of salience within the partitions. The boundary heatmap is presented as a matrix mimicking the shape of the color grid in Fig. 1 where the value in each individual cell represents that chip's *boundary probability* (i.e. likelihood it exists on a category boundary) [6].

*Schematic Similarity:* Schematic Similarity (SS) is a measure that can be used to compare two participants’ color naming data without making any assumptions about the participants’ language or culture. This analysis is based on the partition itself and, therefore, is not dependent on the names assigned to regions of the color space. SS performs term comparisons at the participant level in an effort to preserve maximal information. The range of SS spans from 0 to 1, where two identical schemes have SS = 1 and two completely disjoint schemes have SS = 0.

*Group Error:* The group error function measures the diversity within each WCS language group based on the resulting clusters of participants determined by the mixture model. Error is a function of the number of clusters a language group is split up into and the distance between those clusters. A value of 1 indicates that all participant from a WCS language group were found in distinct clusters while a value of 0 means that all participants from the language were clustered together.

### Software and data access

The *ColorBBDP* software package along with the WCS data used for this project can be accessed publicly (https://github.com/kirbijoe/colorBBDP). The software is written in Python and is cost free and open to all researchers for use.

## Conclusion

We present a methodology for converting color naming data into a form that allows for the discovery of universal patterns by enabling cross-group comparison. This approach eliminates the need for researchers to possess cultural knowledge of language groups, as the data conversion described here retains the structure of the participants’ naming schema. Among the methods described in this paper are new tools for visualizing and analyzing the resulting clusters obtained from the mixture model. Together, these elements comprise the *ColorBBDP* software package—an implemented modeling and analysis of the World Color Survey data set.

## Declaration of Competing Interest

The authors declare that they have no known competing financial interests or personal relationships that could have appeared to influence the work reported in this paper.

## Footnotes

1 The four languages omitted are: Huastec (Language 45), Mampruli (Language 62), Tarahumara-C (Language 92), and Tarahumara-W (Language 93).

2 Color grid is a Mercator projection of the Munsell color space, so the ends of the rows are considered connected but the tops and the bottoms represent the poles of the 3-dimensional solid. Therefore, chips in rows B and I do not have vertically adjacent neighbors in the north and south direction, respectively.
